# Supplementary material for: Restoring protein glycosylation with GlycoShape
Source: Nat Methods. 2024 Oct 14;21(11):2117–27. doi: 10.1038/s41592-024-02464-7 (PMC11541215; doi:10.1038/s41592-024-02464-7)
Supplement: Supplementary file 1 — Supplementary Tables 1–4, Algorithms 1–6 and Figs. 1–4. [file 41592_2024_2464_MOESM1_ESM.pdf]

nature methods

Supplementary information

https://doi.org/10.1038/s41592-024-02464-7

Restoring protein glycosylation with GlycoShape

In the format provided by the authors and unedited

Supplementary Tables

Table 1. Summary of MD simulations conducted to obtain dihedral angles between the glycosidic bonds of the reducing sugar and the glycosylated protein sidechain. For each simulation system, the PDB ID is given where an experimentally-determined protein structure was simulated, and a UniProt ID is given where an AlphaFold structure was simulated.

**Protein**	**PDB/ UniProt ID**	**Glyco-sylated residue(s)**	**Glycosylation types (s)**	**Glycan(s)**	**Simulated time (μs)**
Thromospondon repeat 2	O14514	T369	O-Fucose	Glc(b1-3)Fuc(a1-	1
Glycophorin A	P02724	S63, S66, and T69	O-GalNAc	Neu5Ac(a2-3)-Gal(b1-3) [Neu5Ac(a2-6)]GalNAc(a1-	0.75
3-phosphoinositide-dependent protein kinase 1	1H1W	S92, S105, T222	O-GlcNAc	GlcNAc(b1-	1
Epidermal-like growth factor from Coagulation Factor IX	1EDM	S99 and S107	O-Glucose and O-Fucose	Glc(b1- and Fuc(a1-	1
Epidermal-like growth factor from Coagulation Factor IX	5VYG	S99	O-Glucose	Xyl(a1-3)Xyl(a1-3) Glc(b1-	1
Dystroglycan 1	5GGP	T319	O-Mannose	GlcNAc(b1-2)-Man(a1-	1
Syndecan-1	6EJE	S206	O-Xylose	GlcA(b1-3)GalNAc4S(b1-4) GlcA(b1-3)GalNAc4S(b1-4) GlcA(b1-3)-Gal(b1-3)-Gal(b1-4)Xyl(b1-)	1

TABLE 2. Torsion ranges for different glycosylations

**Residue**	**Sugar**	**Φ Angles**	**Ψ Angles**	**Link Type**
ASN	GlcNAc	(-130, -63)<br>[CG, ND2, C1, O5]	(152, 205)<br>[CB, CG, ND2, C1]	beta
THR	GalNAc	(55, 83)<br>[CB,OG, C1, O5]	(86, 142)<br>[CA, CB, OG, C1]	alpha
THR	Fuc	(274, 304)<br>[CB,OG, C1, O5]	(148, 182)<br>[CA, CB, OG, C1]	alpha
THR	Man	(61, 88)<br>[CB,OG, C1, O5]	(88, 150)<br>[CA, CB, OG, C1]	alpha
THR	GlcNAc	(143, 221)<br>[CB,OG, C1, O5]	(171, 193)<br>[CA, CB, OG, C1]	alpha
SER	GalNAc	(61, 86)<br>[CB, OG, C1, O5]	(163, 224)<br>[CA, CB, OG, C1]	alpha
SER	Fuc	(58, 106)<br>[CB, OG, C1, O5]	(86, 192)<br>[CA, CB, OG, C1]	alpha
SER	Glc	(261, 297)<br>[CB, OG, C1, O5]	(146, 219)<br>[CA, CB, OG, C1]	beta
SER	Xyl	(265, 309)<br>[CB, OG, C1, O5]	(107, 231)<br>[CA, CB, OG, C1]	beta
SER	GlcNAc	(272, 308)<br>[CB, OG, C1, O5]	(178, 300)<br>[CA, CB, OG, C1]	beta
TRP	Man	(110, 150)<br>[CG, CD1, C1, O5]	(-3, 3)<br>[CB, CG, CD1, C1]	alpha

TABLE 3. Overview of the glycoinformatics resources utilised within the GlycoShape platform. For each resource, a description of the information provided for the GlycoShape GDB is tabulated, along with the API address or python module that performs this function.

**GlycoShape GDB information**	**API service / Python module**
Glycam to IUPAC name conversion	GlycoShape GAP
IUPAC to monosaccharide composition	GlycoShape GAP
IUPAC to WURCS name conversion	https://api.glycosmos.org/glycanformatconverter/2.8.2/iupaccondensed2wurcs
IUPAC to GlyTouCan ID fetching	https://api.glycosmos.org/glycanformatconverter/2.8.2/iupaccondensed2wurcs
GlyTouCan ID to biological information	https://api.glygen.org/glycan/detail/
IUPAC to SNFG image creation	glycowork.motif.draw
IUPAC to motif identification	glycowork.motif.annotate
IUPAC to chemical information	glycowork.motif.annotate
IUPAC to termini identification	glycowork.motif.annotate
IUPAC to SMILES name conversion	glycowork.motif.processing
Canonicalize IUPAC name	glycowork.motif.processing
IUPAC to biological information	SugarBase from GlycoWork

Table 4. Default glycan list for uniport annotated PTM information

**Glycosylation Type**	**Glycan Composition**
C-linked (Man) tryptophan	Man
O-linked (Fuc...) serine	Fuc
O-linked (Fuc...) threonine	Fuc
O-linked (GalNAc...) serine	Neu5Ac(a2-3)Gal(b1-3)GalNAc
O-linked (GalNAc...) threonine	Neu5Ac(a2-3)Gal(b1-3)GalNAc
O-linked (Glc...) serine	Glc
O-linked (Glc...) threonine	Glc
O-linked (Man...) serine	Neu5Ac(a2-3)Gal(b1-4)[Fuc(a1-3)]GlcNAc(b1-2)Man
O-linked (Man...) threonine	Neu5Ac(a2-3)Gal(b1-4)[Fuc(a1-3)]GlcNAc(b1-2)Man
O-linked (Xyl...) serine	Xyl
O-linked (Xyl...) threonine	Xyl
O-linked (Xyl...) (chondroitin sulfate) serine	GalNAc(b1-4)GlcA(b1-3)GalNAc(b1-4)GlcA(b1-3)-GalNAc(b1-4)GlcA(b1-3)GalNAc(b1-4)GlcA(b1-3)-Gal(b1-3)Gal(b1-4)Xyl
O-linked (Xyl...) (chondroitin sulfate) threonine	GalNAc(b1-4)GlcA(b1-3)GalNAc(b1-4)GlcA(b1-3)-GalNAc(b1-4)GlcA(b1-3)GalNAc(b1-4)GlcA(b1-3)-Gal(b1-3)Gal(b1-4)Xyl
O-linked (GlcNAc) serine	GlcNAc
O-linked (GlcNAc) threonine	GlcNAc
N-linked (GlcNAc...) asparagine	GlcNAc(b1-2)Man(a1-3)[GlcNAc(b1-2)Man(a1-6)]-Man(b1-4)GlcNAc(b1-4)GlcNAc
N-linked (GlcNAc...) (complex) asparagine	Neu5Ac(a2-6)Gal(b1-4)GlcNAc(b1-2)Man(a1-3) [Neu5Ac(a2-6)Gal(b1-4)GlcNAc(b1-2)Man(a1-6)]-Man(b1-4)GlcNAc(b1-4)[Fuc(a1-6)]GlcNAc
N-linked (GlcNAc...) (hybrid) asparagine	Neu5Ac(a2-6)Gal(b1-4)GlcNAc(b1-2)Man(a1-3) [Man(a1-3)[Man(a1-6)]Man(a1-6)]Man(b1-4)Glc-NAc(b1-4)GlcNAc
N-linked (GlcNAc...) (high mannose) asparagine	Man(a1-3)[Man(a1-6)]Man(a1-6)[Man(a1-3)]-Man(b1-4)GlcNAc(b1-4)GlcNAc

Supplementary Algorithms

Algorithm 1. Steric Interaction Scoring Function

Steric Interaction Calculation

Steric (Garr, Parr):

initialize r **to** 0
calculate euclidean distances D between G **and** P using `cdist`
select distances C from D where distance < 1.7
for each distance i **in** C[2**:**−1]**:** // Ignoring H & "d"
    increment r by $200 \times \exp(-i^2)$
return r

Algorithm 2. Fitness Function for Evaluating Steric Interactions Post-Rotation

Fitness Function

Fitness F (P, G, φ,ψ):

define function `rotate` with parameters G, φ, ψ, a, b, c, d
apply `rotate` **to** G **to** obtain G'
invoke `Steric` function with G' **and** P **to** compute steric interactions
assign the output of `Steric` function **to** variable **steric**
return steric

Algorithm 3. Algorithm for Rotating Molecular Group G Using Quaternion Rotation Matrices

Group Rotation Using Quaternion Matrices

rotate (G, phi, psi, a, b, c, d):

initialize $q_{\{\varphi\}}$ as quaternion representing rotation by φ around axis $b - c$
initialize $q_{\{\psi\}}$ as quaternion representing rotation by ψ around axis $c - d$
for each atom x **in** G**:**
    calculate new position x' by applying $q_{\{\varphi\}}$ **then** $q_{\{\psi\}}$ **to** x
    update position of x **in** G **to** x'
return modified G as G'

Algorithm 4. Torsion Pairs Identification Algorithm

Torsion Pairs Identification in Molecular Structures

```
torsion_pairs (G, connect):
let empty list cycles
for each cycle in nx.cycle_basis(G):
    return add atoms in cycle to cycles
let empty list pairs
for each node in "G":
    if degree of node == 2 and node not in cycle:
        find neighbors j, k of node
        if degree of j > 1:
            find neighbors l of j excluding node
            pairs.append([l[0], j, node, k])
        if degree of k > 1:
            find neighbors m of k excluding node
            pairs.append([m[0], k, node, j])
return pairs
```

Algorithm 5. Algorithm for Generating New Conformations by Random Torsion Rotation

Random Torsion Rotation for Molecular Conformations

```
wiggle (G, pairs):
let angle_range be (-10, 10) degrees
let G_new be a copy of G
for each pair in pairs:                                   // from torsion_pairs
    let [a, b, c, d] be atoms in the torsion pair
    let random_angle be a random value from angle_range
    rotate G_new at torsion defined by [a, b, c, d] by
    random_angle using quaternion rotation
return G_new
```

ALGORITHM 6. Genetic Algorithm for Optimization of Molecular Conformations

Genetic Algorithm for Minimizing the Fitness Function

genetic_algorithm (P, G, $[\varphi_{\text{range}}]$, $[\psi_{\text{range}}]$):

let population_size be 128
let mutation_rate be 0.2
let generations be 8
let individial$_{\text{best}}$ be φ_{best} **and** ψ_{best} with fitness$_{\text{best}}$ 100000
let population be an empty list of size population_size
for each individual **in** population**:**
    let $\varphi_{\text{individual}}$ be a random number within $[\varphi_{\text{range}}]$
    let $\psi_{\text{individual}}$ be a random number within $[\psi_{\text{range}}]$
for gen from 1 **to** generations**:**
    for each individual **in** population**:**
        let individual [fitness] be F(P, G, $\varphi_{\text{individual}}$, $\psi_{\text{individual}}$)
    sort population by increasing fitness
    let parents be the top 50% of population
    let children be crossover_and_mutate(parents, mutation_rate)
    replace_worst_with_children(population, children)
    if fitness of the first individual **in** the sorted population $<$ fitness$_{\text{best}}$
        let best$_{\text{individial}}$ be the first individual **in** the sorted population
return individial$_{\text{best}}$ (φ_{best} **and** ψ_{best})

Supplementary Figures

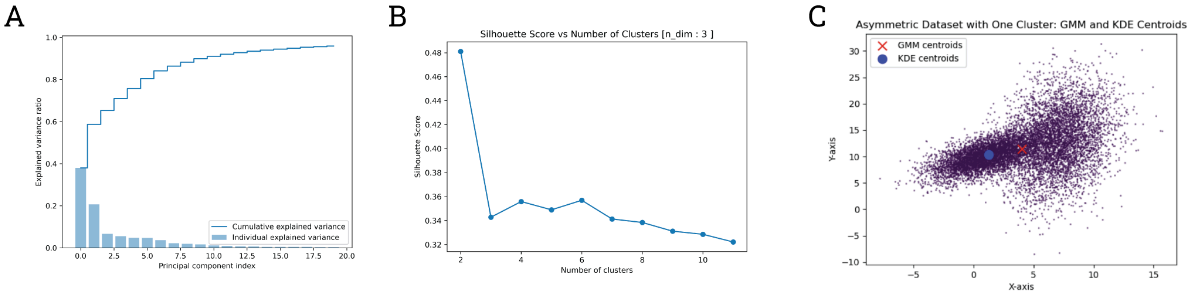

Figure 1. **A)** Cumulative and individual explained variance by principal components. **B)** Silhouette score analysis for determining the optimal number of clusters. The plot depicts the silhouette scores for different numbers of clusters, with the peak at 6 clusters suggesting it as the optimal count for the given dataset. **C)** Comparison of centroids determined by Gaussian Mixture Model (GMM) and Kernel Density Estimation (KDE) in an asymmetric dataset. The scatter plot visualizes the data points with the GMM centroids (marked as 'X') and KDE centroids (marked as circles), showcasing the difference in centroid location due to the asymmetric distribution of the data.

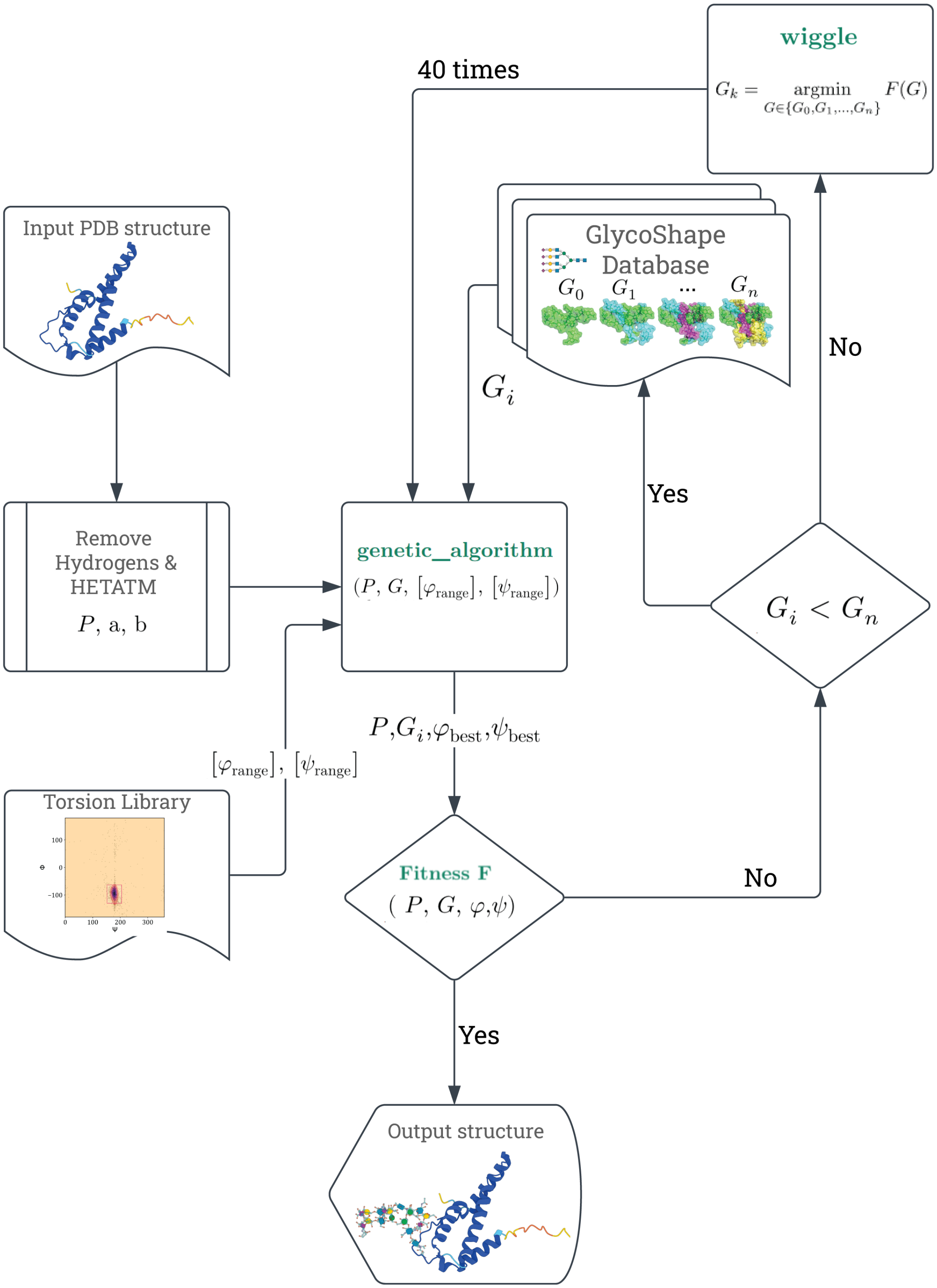

FIGURE 2. Re-Glyco algorithm flowchart

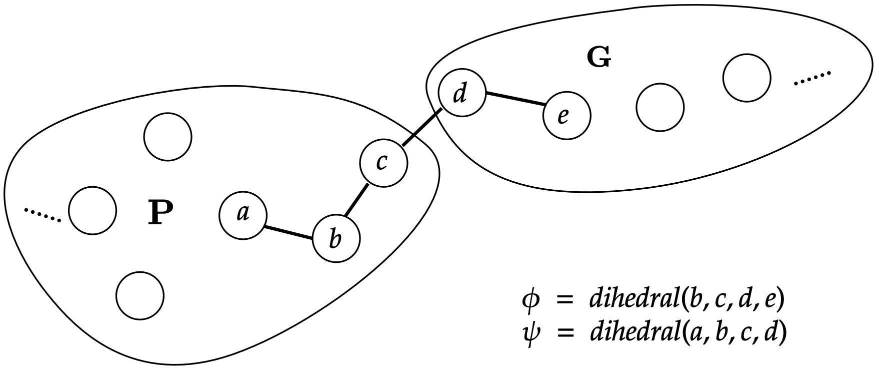

FIGURE 3. Torsion Angles(φ, ψ) between the protein(P) and the glycan(G).

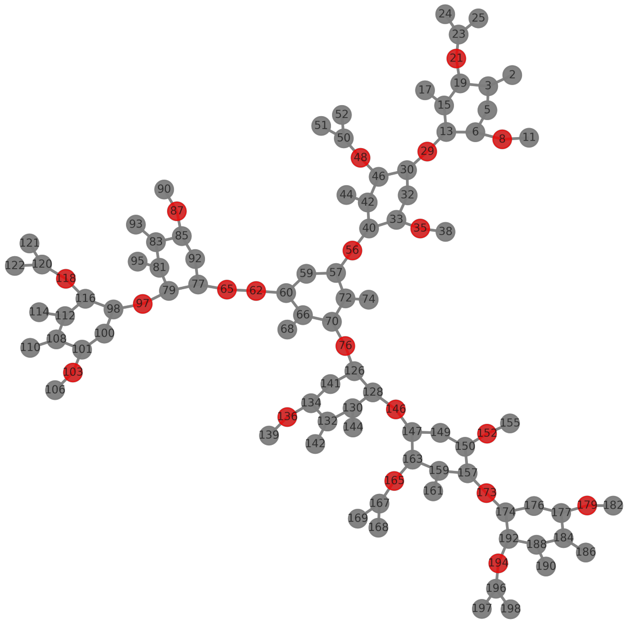

FIGURE 4. Identified torsion pairs in a glycan(G) using networkX package.
